# Research on biomimetic design and impact characteristics of periodic multilayer helical structures

**DOI:** 10.3389/fbioe.2023.999137

**Published:** 2023-04-07

**Authors:** Yu-Xi Liu, Ai-Hua Li, Shi-Yun Lin, Hong Sun, Bin Chen

**Affiliations:** ^1^ School of Smart Health, Chongqing College of Electronic Engineering, Chongqing, China; ^2^ Department of Gastroenterology, Chongqing University Cancer Hospital, Chongqing, China; ^3^ Green Aerotechnics Research Institute of Chongqing Jiaotong University and School of Aeronautics, Chongqing Jiaotong University, Chongqing, China; ^4^ College of Aerospace Engineering, Chongqing University, Chongqing, China

**Keywords:** periodic helical structure, biomimetic composite, osteon, mineralized collagen fibril, impact characteristic

## Abstract

Osteons are composed of concentric lamellar structure, the concentric lamellae are composed of periodic thin and thick sub-lamellae, and every 5 sub-lamellae is a cycle, the periodic helix angle of mineralized collagen fibers in two adjacent sub-lamellae is 30°. Four biomimetic models with different fiber helix angles were established and fabricated according to the micro-nano structure of osteon. The effects of the fiber periodic helical structure on impact characteristic and energy dissipation of multi-layer biomimetic composite were investigated. The calculation results indicated that the stress distribution, contact characteristics and fiber failur during impact, and energy dissipation of the composite are affected by the fiber helix angle. The stress concentration of composite materials under external impact can be effectively improved by adjusting the fiber helix angle when the material composition and material performance parameters are same. Compared with the sample30, the maximum stress of sample60 and sample90 increases by 38.1% and 69.8%, respectively. And the fiber failure analysis results shown that the model with a fiber helix angle of 30° has a better resist impact damage. The drop-weight test results shown that the impact damage area of the specimen with 30° helix angle is smallest among the four types of biomimetic specimens. The periodic helical structure of mineralized collagen fibers in osteon can effectively improve the impact resistance of cortical bone. The research results can provide useful guidance for the design and manufacture of high-performance, impact-resistant biomimetic composite materials.

## Graphical Abstract

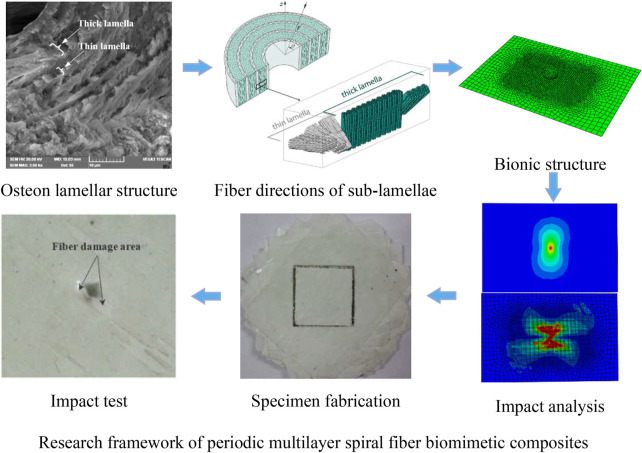

## Introduction

Bone is a highly optimized composite material with outstanding mechanical properties, which has a very complex structure and is organized at different levels (Wang et al., 2020; Ingrole et al., 2021). Composite materials with high strength, light weight (Rahimizadeh et al., 2021) and impact resistance (Wang et al., 2021; Wang et al., 2022) have a wide range of needs in the fields of aerospace, military, vehicles and other fields (Dong et al., 2022; Sharma et al., 2022). Therefore, the research of biomimetic composites can provide inspiration for the design of materials with excellent mechanical properties and meet the special requirements of engineering (Bhudolia and Joshi, 2018; Jiang et al., 2019; Alizadeh and Ebrahimzadeh, 2022).

**FIGURE 1:**
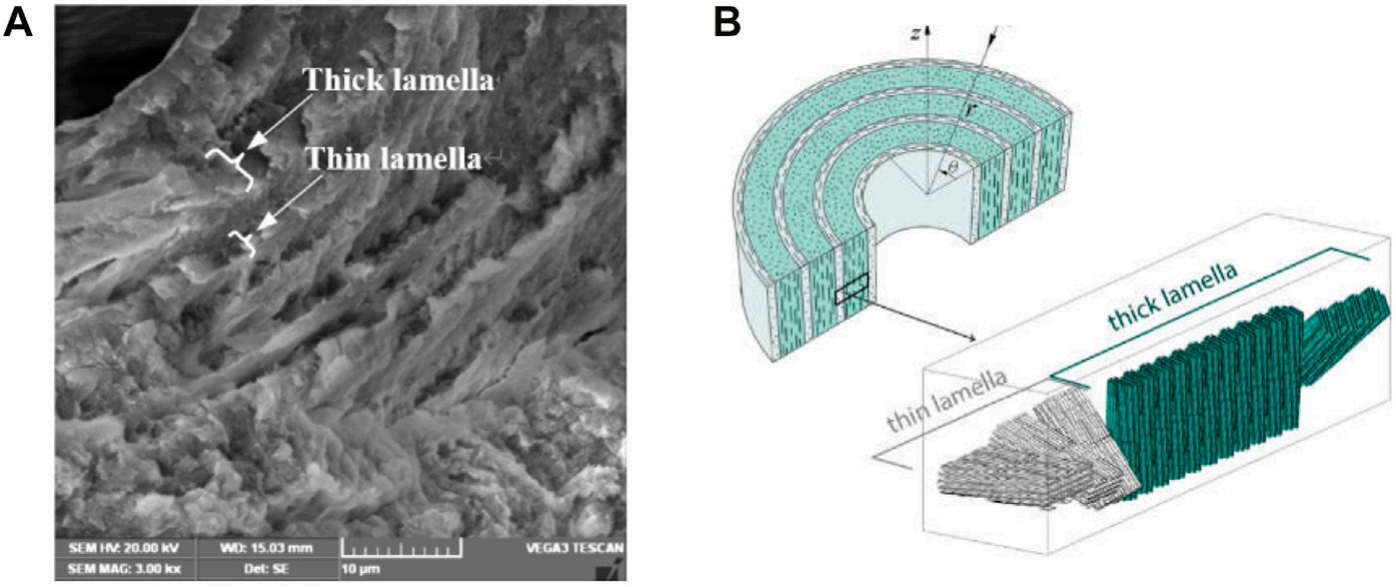
Lamella structure of osteon. **(A)** Thin and thick lamellae of osteon, **(B)** arrangement direction of fibers in adjacent lamellae.

Lamellar bone is the most abundant type in the cortical bones and composed of osteonal tissue. Osteons are cylindrical shaped structural and is composed of concentric lamellar structure (Figure 1A) (Liu et al., 2017), the diameter ranges from 50 to 500 μm (Currey, 2012), and the lamella thickness is about of 3–7 μm (Giner et al., 2014a). Cortical bone is mainly composed of organic phase (Fratzl and Fratzl, 2010) and inorganic phase, and the organic phase is mainly formed by mineralized collagen fibers (Hamed et al., 2010). Xu et al. (Xu et al., 2003) characterized the micro-mechanical properties of human lamellar bone, and the results showed that the elastic modulus of thick lamellar bone was higher than that of thin lamellar bone. Gupta et al. (Gupta et al., 2006) investigated the microstructure of osteon, and the results showed that the osteon consists of a laminated cylindrical composite composed of mineralized collagen fibers. Carnelli et al. (Carnelli et al., 2013) evaluated the elastic constants of the sublayers of mineralized collagen fibrils in osteonal lamella, and the results show that the hierarchical structure of lamellar bone was the main determinant of the adjustment of tissue mechanical properties. Reznikov et al. (Reznikov et al., 2013) investigated the orientation of average collagen array and the dispersion of local collagen fibers, and three different sub-lamellar structural motifs. Varga et al. (Varga et al., 2013) investigate the 3D organization of mineralized collagen fibrils in human cortical bone, and find two specific dominant patterns, oscillating and twisted ply-woods coexisting in a single osteon, and the orientation of collagen fibrils changed periodically. Weiner et al. (Weiner et al., 1997) measured the angle between adjacent arrays of rat bone lamellae, the results shown that most of the angles were about 30°. The mineralized collagen fibers in osteons have their own uniqueness, which are periodic helical arranged and every 5 sub-lamellae constitute a lamella, the offset angle of fibers in two adjacent sub-lamellae is 30° (Giraudguille, 1988; Liu et al., 2000). Giner et al. (Giner et al., 2014a) drew a schematic diagram of the staggered structure of sub-lamellae of osteons and the fiber directions of the five sub-lamellae (Figure 1B), and the 5 sub-layers are simplified to a thin layer and a thick layer (Vercher et al., 2014).

At present, the existing literatures have reported the mechanical properties and structure bionics of multilayer fiber reinforced composites from different perspectives (Jansen et al., 2016; Rua et al., 2021), the research on osteon mainly focuses on microstructure (Giner et al., 2014b; Reznikov and Weiner, 2014; Yin et al., 2021), distribution of collagen fibers and osteocyte lacunae (Liu et al., 2017; Liu et al., 2019). However, biomimetic composite materials with periodic helical arrangement of fibers are rarely reported. Based on the distribution of fibers in osteon, four kinds of biomimetic composite model with different helix angle were constructed. In order to compare and analyze the impact characteristics of composite materials affected by the periodic helical arrangement of fibers, four biomimetic composite models were fabricated. Furthermore, the effects of the fiber periodic helical structure on impact characteristic and energy dissipation of multi-layer biomimetic composite were investigated by progressive damage analysis and drop-weight test.

## Material and methods

### Structure biomimetic and impact analysi*s*

A biomimetic composite model was constructed (Table 1, Sample 30) based on the helical structure of mineralized collagen fiber in osteon, and the offset angle of periodic helix fiber is 30°. In addition, for comparing and analyzing the effects of fiber helix angle on the impact resistance of biomimetic composites, three composite material models of osteon-like with 0°/90° model and fiber helix angle of 15° and 60°models were constructed (Table 1). Then, the effects of fiber arrangement structure on the impact resistance and energy dissipation capacity of the bionic composite were investigated based on finite element (FE) analysis.

**TABLE 1:** Models of multilayer fiber biomimetic composites.

Bionic models	Fiber laying mode
Small helix angle (Sample15)	[0/15/30···/165]
Medium helix angle (Sample30)	[0/30/60···/150]_2s_
Large helix angle (Sample60)	[0/60/120]_4s_
Orthogonal m (Sample90)	[0/90]_6s_

**FIGURE 2:**
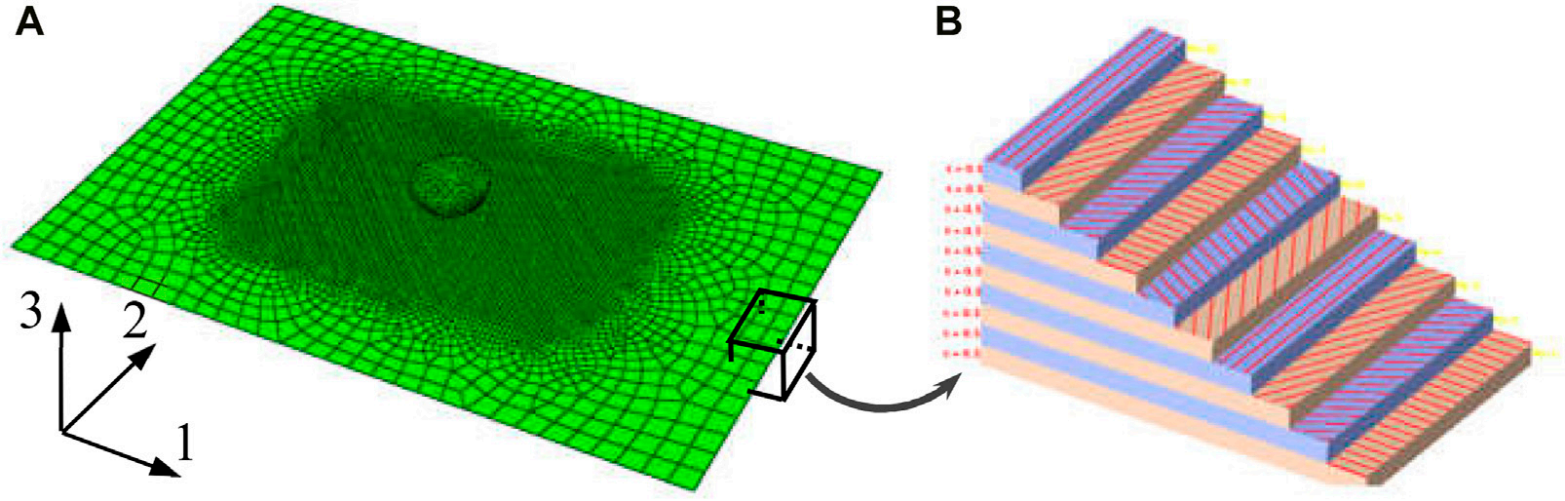
Impact analysis model of biomimetic composites. **(A)** Finite element model for impact analysis, **(B)** fiber arrangement direction of composite material (red indicates fiber).

According to the periodic helical arrangement structure of the fibers in osteon, a 12-layer periodic helical structure bionic model was constructed in ABAQUS (Figure 2), and it is assumed that the thickness of each sublayer in the helical structure is same and the layers are well integrated among themselves. The geometric size of the model is 150 mm × 100 mm×6 mm (standard thickness in ASTM-D-7136: 4–6 mm), the number of layers is 12, and the thickness of each sublayer is equally distributed as shown in Table 1. The mechanical performance parameters of the composite material models used in FE analysis are shown in Table 2 (Giraudguille, 1988; Weiner et al., 1997; Hansen and Martin, 1999; Liu et al., 2000; Karakuzu et al., 2010; Giner et al., 2014b; Reznikov and Weiner, 2014; Vercher et al., 2014; Jansen et al., 2016; Liu et al., 2019; Rua et al., 2021; Yin et al., 2021; Ekhtiyari and Shokrieh, 2022).

**TABLE 2:** Characteristic parameters of composite material.

Material characteristics	Value	Material characteristics	Value
Density*ρ*/(kg/m3)	1830	Transverse compressive strength*Y*_c_/MPa	124
Longitudinal modulus*E*_11_/GPa	40.51	In-plane shear modulus *G*_12_/GPa	3.1
Transverse modulus*E*_22_/GPa	13.96	In-plane shear strength, *S*_12_/GPa	69
Poisson’s ratio*ν*_12_	0.22	Interlaminar shear strength, *S*_1_/MPa	38
Longitudinal tensile strength*X*_t_/MPa	783.3	Longitudinal critical energy release rate *G*_cr,L_/(kN·m^-1^)	40
Longitudinal compression strength*X*_c_/MPa	298	Transverse critical energy release rate *G*_cr,T_/(kN·m^-1^)	0.3
Transverse tensile strength*Y*_t_/MPa	64		

Drop-weight impact analysis was conducted on the four bionic models. The impact energy of 20 J was selected, the mass of the drop-weight is 2kg, and the critical contact velocity is 4.47 m/s 45# steel is selected as the punch material, the elastic modulus *E* and Poisson’s ratio *ν* of the punch are 210GPa and 0.3, respectively. The shape of the punch tip is hemispherical with a diameter of *ϕ*16 mm, and the punch is constrained as a rigid body. A reference point was chosen on the punch and a mass point was added, and impact velocity was applied on the mass point. The impact energy is applied to the punch according to formula *E* = *mv* ^2^/2.

The analysis model of periodic helical bionic structure with fiber helix angle of 30° is shown in Figure 2A, the distribution direction of the fibers in each layer are shown in Figure 2B. The four models constructed in this analysis are modeled by 3D shell element for obtaining higher accuracy, and the element type and size are the same. Furthermore, due to the severe mesh deformation in impact center area, the mesh in the impact center area was refined for improving the calculation accuracy. Then, the mesh size was increased gradually from the middle region to the boundary region to ensure the mesh quality and reduce the calculation time. Through grid optimization, the mesh information is 6 292 nodes and 6 275 hyperbolic shell elements (S4R) with large strain, reduced integral and sand leakage control. Full restraint was applied to the four sides of the model based on the drop-weight test requirements of ASTM-D-7136 for. Due to the hard contact between the punch and the composite plate in impact process which will cause the failure of contact mesh, thus the ordinary hard contact algorithm is adopted. The failure degradation of the mesh is based on the Hashin failure criterion, and the fiber is 0° along the 1-direction and 90° along the 2-direction (Figure 2A).

### Material failure criterion of impact analysis

The damage types of multilayer composite materials are mainly divided into in-plane damage and interlaminar damage under low-speed impact load. The in-plane damage of composites mainly includes fiber fracture and matrix crack, and the interlaminar damage mainly refers to delamination failure between sublayers. Energy-based damage evolution was adopted for the interlaminar interface of the multi-layer composite. The impact process of composite materials can be roughly divided into four stages: compression stage, shear stage, fiber stretching deformation stage and penetration stage (Doddamani et al., 2023). Because the material is squeezed under impact load. Therefore, this paper focuses on the comparison and analysis of the in-plane damage of materials with different fiber arrangement methods, and the interlaminar damage of composite materials was ignored.

### Damage failure criterion

The Hashin failure criterion can accurately determine various damage failure modes and is simple and effective, it has been widely used in practice. The combination of Hashin failure criterion and stiffness degradation criterion can simulate the progressive damage process of composite materials and can be easily realized. Thus, the Hashin criterion was used to simulate the impact damage of multilayer bionic composite. The expressions of Hashin failure criterion are as follows (Hashin and Rotem, 1973):

Fiber tensile failure ( $\sigma_{11}\geq 0$ )

$${\left(\frac{\sigma_{11}}{X^{T}}\right)}^{2}+{\left(\frac{\sigma_{12}}{S^{L}}\right)}^{2}=1$$ (1)

Fiber compression failure ( $\sigma_{11}<0$ )

$${\left(\frac{\sigma_{11}}{X^{C}}\right)}^{2}=1$$ (2)

Tensile failure of matrix ( $\sigma_{22}\geq 0$ )

$${\left(\frac{\sigma_{22}}{Y^{T}}\right)}^{2}+{\left(\frac{\sigma_{12}}{S^{L}}\right)}^{2}=1$$ (3)

Matrix compression failure ( $\sigma_{22}<0$ )

$${\left(\frac{\sigma_{22}}{2S^{T}}\right)}^{2}+\left[{\left(\frac{Y^{C}}{{2S}^{T}}\right)}^{2}-1\right]∙{\left(\frac{\sigma_{22}}{Y^{C}}\right)}^{2}+{\left(\frac{\tau_{12}}{S^{L}}\right)}^{2}=1$$ (4)

where, *X* ^*T*^ is the longitudinal tensile strength of the single layer; *X* ^*C*^ is the longitudinal compressive strength of the single layer; *Y* ^*T*^ is the transverse tensile strength of the single layer; *Y* ^*C*^ is the transverse compressive strength; *S* ^*L*^ is the longitudinal shear strength; *S* ^*T*^ is the transverse shear strength; *σ*_11_, *σ*_22_ and *τ*_12_ are effective stress tensor components.

### Material degradation criterion

The failure process of multilayer fiber reinforced composites is a complex process of progressive deterioration. At the initial stage of loading, some form of damage will occur in the weak part of the composite and cause the redistribution of load, but it may not be manifested macroscopically. With the increase of load, the damage accumulation and superposition, causing the continuous degradation of composite material properties and the continuous decrease of bearing capacity, until the whole laminates are destroyed. The progressive failure analysis method considers the local damage through the material performance degradation model, which can better simulate the failure mechanism, interaction and propagation process, and the ultimate failure load of composite laminates.

Material degradation means that when the mesh satisfies certain failure criteria in the FE models, the meshes in the model will be damaged. According to these different damage modes, the material properties of the damage element in the model need to be given new values according to certain rules, so as to obtain a new material model. In the FE progressive damage analysis, there are many methods to degrade the stiffness of the mesh. The material parameter degradation mode (Zhang and Chen, 2021) was used to degrade the stiffness of damaged area in this research, and different in-plane damage modes correspond to different degradation schemes, as shown in Table 3.

**TABLE 3:** Material stiffness degradation criterion.

Damage mode	Degradation criteria
Matrix tensile failure (*σ*_22_ ≥ 0)	*Q*_0_ = 0.2*Q* (*Q* = *E*_22_, *G*_23_, *ν*_12_)
Matrix compression failure (*σ*_22_ < 0)	*Q*_0_ = 0.4*Q* (*Q* = *E*_22_, *G*_23_, *ν*_12_)
Fiber tensile failure (*σ*_11_ ≥ 0)	*Q*_0_ = 0.07*Q* (*Q* = *E*_11_, *G*_23_, *ν*_12_)
Fiber compression failure (*σ*_11_ < 0)	*Q*_0_ = 0.2*Q* (*Q* = *E*_11_, *G*_23_, *ν*_12_)

### Biomimetic composite fabrication and test

#### Specimen fabrication

Unidirectional glass fiber prepreg (glass fiber/epoxy resin, model: G 12,500) was used to fabricate bionic spiral structure composite laminates. The fiber ratio of unidirectional glass fiber prepreg is 125 g/m^2^, the resin content is 33% (included 33wt% - of resin), and the thickness of single layer is 0.1 mm. The fabrication processes of the composite laminate with fiber periodic helix ply structure are as follows.

(1) The prepreg was cut into a square of 120 mm × 120 mm.
(2) The prepreg was arranged periodically at the angles of 15°, 30°, 60° and 90°. The number of layers is 12.
(3) The prepreg lamination is put into the hot press mold, and the hot press (hot press model: hy61zf) is used for hot press curing.

The hot-pressing process is as follows.

(1) The initial pressure is 2t, and the temperature is raised to 200 °C;
(2) After holding the pressure for 5 min, the pressure dropped to 1t;
(3) The four kinds of bionic composite laminate can be obtained by keeping the pressure for 120 min and cooling naturally.

### Drop-weight test

The biomimetic composite laminates were cut into drop-weight impact test specimens (size: 50 mm × mm). Then, the drop-weight impact tests were conducted according to the test method of ASTM- D-7136. The model of impact testing machine is XH-2000, and the manufacturer is Yangzhou Xinhong Test Machine Factory. The diameter of rigid body punch is 8.5 mm and the dimension of punch tip is 4mm, the mass of the drop-weight is 2kg, the drop-height is 1 m. The total impact energy (J) of a specimen was computed by the equation *E*= *MgH*, where *M* is the weight of the hammer (kg), *g* is 9.8 m/s^2^, and *H* is drop-height (m).

## Results and discussion

### Effects of laying mode on stress distribution

**FIGURE 3:**
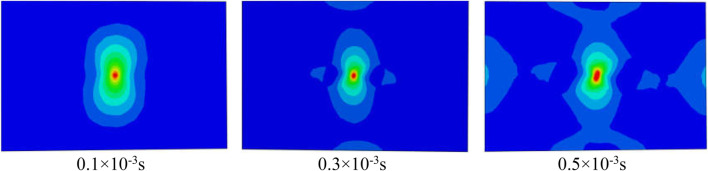
The nephogram of Mises stress distribution at the typical time moment of Sample30 when the impact energy is 20 J.

Figure 3 shows the nephogram of Mises stress distribution at the typical time moment of Sample30 biomimetic model under the impact energy of 20 J. From this stress distribution nephogram can be seen, the stress wave is transmitted in the direction of the flat plate surface and the direction perpendicular to the flat plate surface from the impact center area in the composite laminate. When the stress exceeds its limit value, the mesh will fail and be destroyed. In addition, from the stress distribution of the biomimetic model with helix angle of 30° (Sample30), the Mises stress contour line roughly shows a “peanut” shape, which is consistent with the results of the general composite impact test (Chang and Lessard, 1991), which indicates the correctness of the numerical model and analysis calculation established in this paper.

**FIGURE 4:**
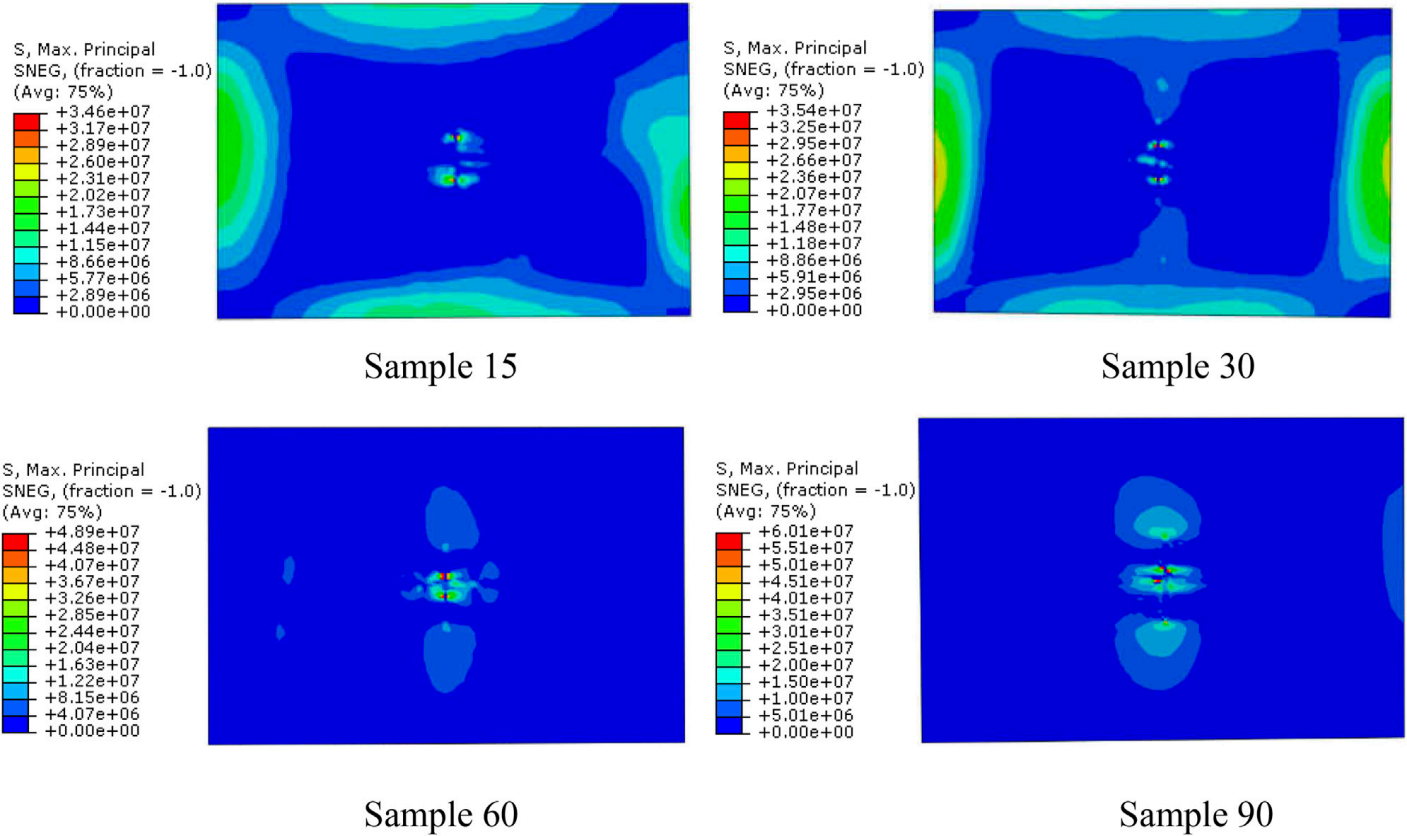
Nephogram of the maximum principal stress distribution of four biomimetic composite models when the impact energy is 20 J.

Figure 4 shows the maximum stress distribution clouds of four biomimetic models based on the node maximum principal stress principle when the impact energy is 20 J. It can be seen from the analysis results that with the increase of the fiber helix angle, the shape of the stress contours and the maximum stress have obvious differences. The maximum stresses of Sample15, Sample30, Sample60, and Sample90 are 34.6 MPa, 35.4 MPa, 48.9 MPa, and 60.1 MPa, respectively. The maximum stresses of Sample15 and Sample30 are similar, but with the increase of the helix angle, the concentration of stress obviously increases. Compared with the Sample30 model, the maximum stress of Sample60 and Sample90 models increases by 38.1% and 69.8%, respectively.

The above analysis results indicated that for fiber-reinforced composite materials, when the material composition and material performance parameters are same, the mechanical properties of the material can be effectively improved and the stress concentration of the composite material when subjected to external impact can be reduced by adjusting the laying way of the fibers. For periodic fiber helical lamination composite materials, a smaller helix angle helps to reduce the stress concentration.

### Effects of laying mode on impact characteristics

The maximum contact force and damage behavior are significantly different when the multi-layer composites with different fiber arrangement are subjected to external impact. For comparing and analyzing the effects of fiber arrangement methods on impact characteristics, the same boundary conditions were used in the four models and the impact damage analysis was conducted.

### Contact characteristics during impact

**FIGURE 5:**
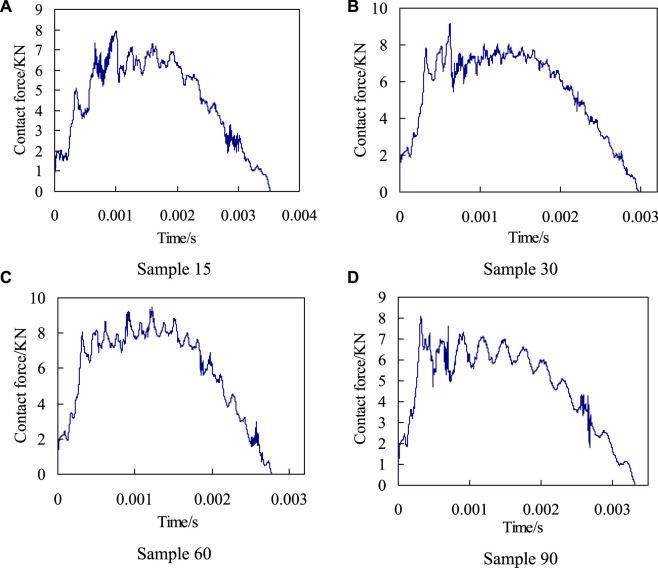
The curves of impact force-time history when the impact energy is 20 J. **(A)** Sample 15, **(B)** Sample 30, **(C)** Sample 60, and **(D)** Sample 90.

When the impact energy is 20J, the impact load variation history of the four biomimetic composite models with time are shown in Figure 5. According to the analysis results, during the impact process, the time history of the impact load can generally be divided into two stages: the first stage is stamping stage, where the impact load continuously increases and finally reaches the peak point of the impact load; the other stage is rebound stage, in which the impactor is rebounded and gradually detaches from the composite laminate. The impact load gradually attenuates until the impactor completely detaches from the surface of the plate, and the impact load decreases to 0.

By comparing and analyzing the change history of contact load over time of four biomimetic models (Figure 5), it is can be seen that there are significant differences in the change process and peak value of contact load during the impact process. And there are also differences in the timing of the peak point of contact load for the four models. The Sample90 model first appears the peak point, and the Sample60 finally appears the peak point.

**FIGURE 6:**
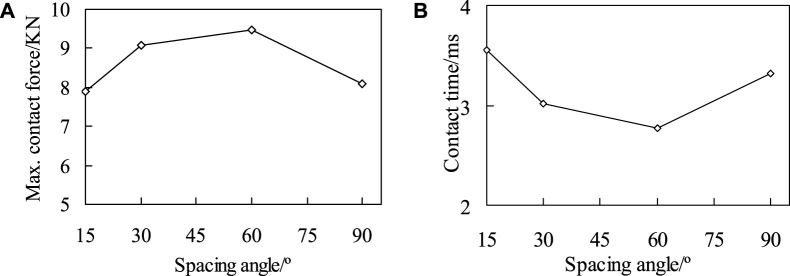
The maximum contact force **(A)** and contact time **(B)** of four different models when the impact energy is 20 J.

The contact load peak curve and impact contact time for the four models are shown in Figure 6. It can be seen from Figure 6B that with the increase of the fiber helix angle (from 15° to 60°), the contact time decreases significantly, and the impact contact time of Sample60 decreases by 22.1% compared to Sample15. The impact contact characteristics of the four models indicate (Figure 6) that the fiber arrangement methods directly affect the peak contact load and contact time, and there are significant differences between the fiber helical structure and the orthogonal arrangement structure. In addition, according to the maximum principal stress analysis results of the four models can be known (Figure 4), the impact stress of Sample90 is the largest, and the stress concentration phenomenon is the most obvious, which means that damage occurs first during the impact process. The analysis results of the impact contact characteristics of the above four models also show that, when the material composition and performance parameters are same, the impact resistance characteristics of the composite plate can be effectively improved by adjusting the fiber laying method. With the reduction of fiber helix angles, its stress distribution becomes more uniform, and the stress concentration phenomenon and impact resistance characteristics are significantly improved.

### Fiber failure during impact

**FIGURE 7:**
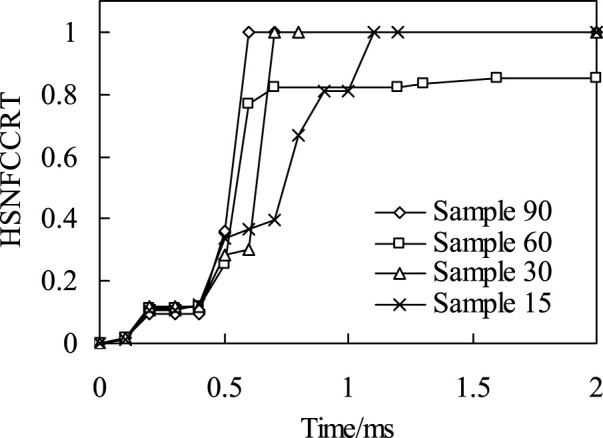
The relationship of impact time history and fiber compression failure.

**FIGURE 8:**
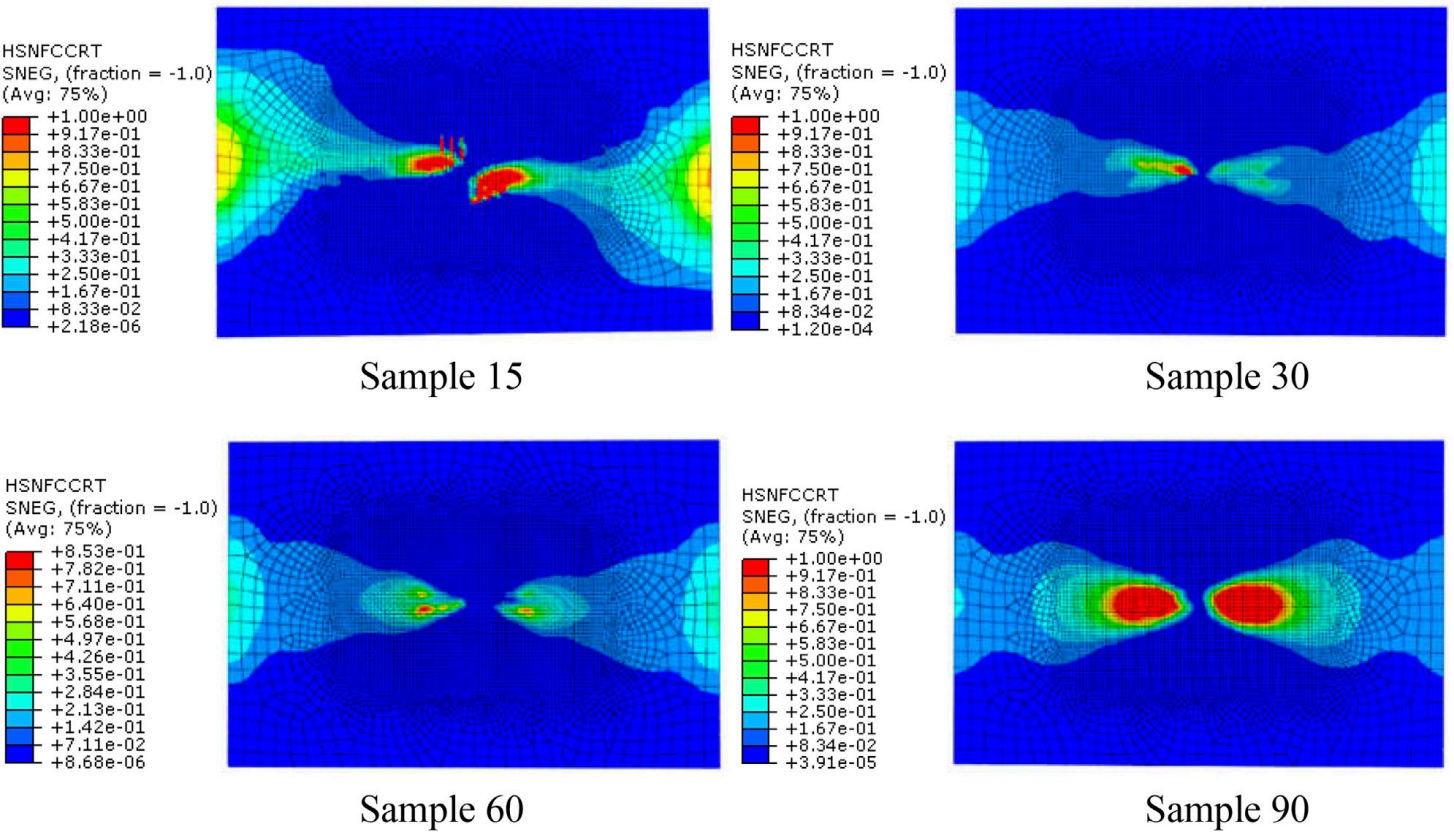
The fiber compression failure distribution when the impact energy is 20 J (the red area represents the part of fiber compression failure).

*Fiber compression failure*. When the impact energy is 20J, the initial failure of fiber compression and the failure distribution nephogram of the four kinds of bionic composites in the impact process were shown in Figures 7, 8, respectively. According to the fiber compression failure criterion, the fiber compression failure occurs when the failure criterion is greater than or equal to 1. It can be seen from the analysis results (Figure 7) that with the increase of impact energy, the fiber compression failure occurs first in the sample 90, followed by the sample 30 model, and finally the sample 15 model. However, there is no obvious fiber compression failure in the sample 60 model under the impact load. In addition, it can be seen from Figure 5 that although the fiber compression failure exists in the sample15, sample30 and sample90 models, there is a significant difference in the failure initiation time. It can be seen from the failure distribution nephogram (Figure 8) that the fiber compression failure ratio in the sample90 model is the largest, followed by the sample15 model, and the failure proportion in the sample30 model is the smallest, which indicates that the collagen fiber arrangement structure with a helix angle of 30° is helpful to enhance the compression resistance ability of osteon.

The above analysis results show that the fiber stacking mode directly affects the compression failure of the fiber. By adjusting the fiber stacking mode in the multi-layer composite, the initial time of fiber compression failure and the ratio of fiber compression failure can be effectively improved.

**FIGURE 9:**
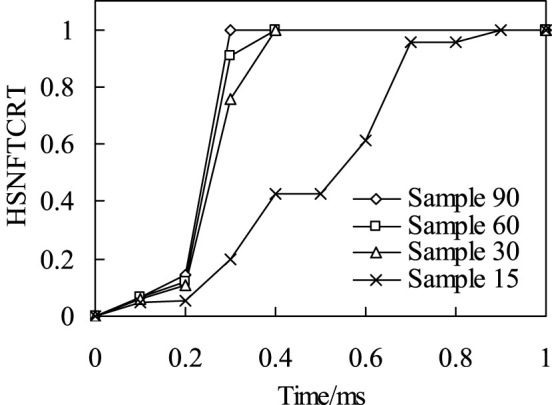
The relationship of impact time history and fiber tensile failure.

*Fiber tensile failure*. The time history of fiber tensile initial failure of four bionic composite models during impact process was shown in Figure 9. It can be seen from the analysis results that under the same impact load, the model of sample90 first appears fiber tensile failure. The second model is sample60 and sample30, but there are some differences in their time histories. Finally, the sample15 model, and the fiber tensile failure time of sample15 is much later than the first three models, which shows that the fiber has strong ability to resist tensile crack initiation in sample 15. Followed by the sample60 and sample30 models, but there are also certain differences in their time history. Finally, the sample15 model, and the fiber tensile failure time of sample15 is much later than the first three models, which shows that the fiber has strong ability to resist tensile crack initiation in model sample15.

**FIGURE 10:**
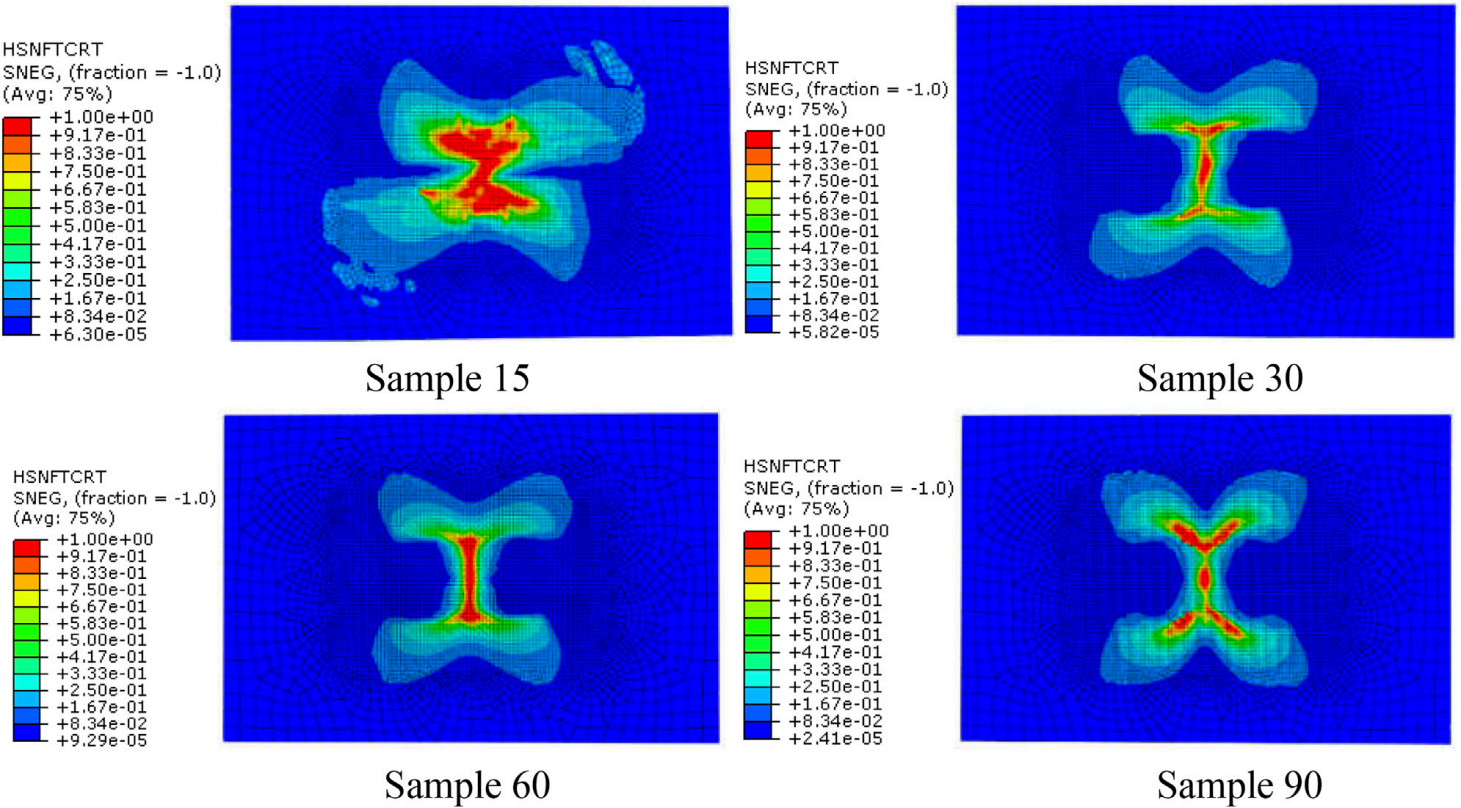
The fiber tensile failure distribution when the impact energy is 20 J.

It can be seen from the distribution nephogram of fiber tensile failure (Figure 10) that the proportion of fiber tensile failure increases in three models with fiber helix angle from 30° to 90°. However, the fiber tensile failure ratio is the largest when the helix angle is 15°, which indicates that the smaller the helix angle, the stress of the fiber in the model is more uniform. Once the failure occurs, the damage ratio will increase rapidly. The above analysis results show that the tensile strength of the multi-layer composite with fiber helix angle of 30° is stronger. Simultaneously, it also shows that the helix structure of collagen fibers with a helix angle of 30° in osteon helps to enhance the tensile strength of cortical bone.

### Effects of laying mode on energy dissipation

Energy is an important factor in low-speed impact test. The difference between the impact energy and the kinetic energy of punch pin at the end of the impact is defined as the energy dissipated. The “energy dissipated” defined here also includes other energy consumption during impact process, such as strain energy released by sandwich plate, kinetic energy of sandwich plate, strain energy released by punch, consumption of viscous damping and friction. In this study, the energy dissipation capacity of impact kinetic was mainly compared and analyzed. The calculation method of impact energy as follows: the kinetic energy of the punch at the moment of contact is regarded as the impact energy. Therefore, the actual impact energy *E*_*impact*_ and the dissipated energy of composite plate *E*_*dissipated*_ are defined as follows:

$$E_{impact}=\frac{1}{2}{mv}_{0}^{2}$$ (5)

$$E_{dissipated}=\frac{1}{2}{mv}_{0}^{2}-\frac{1}{2}{mv}_{t}^{2}$$ (6)

where, *m* is the mass of the punch, *m* = 2 kg in this study (the mass of different sizes of punch is slightly different in actual experiment); *v*_0_ and *v*_*t*_ are the velocities of the punch, at the moment of contact and separation between the punch and the upper surface of the model, respectively.

**FIGURE 11:**
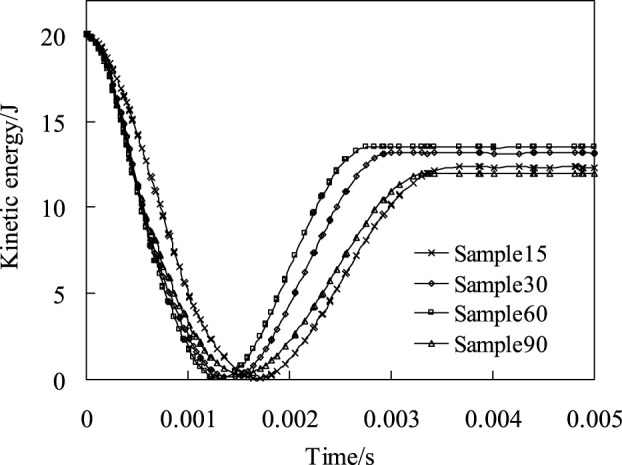
The change history of kinetic energy of the four models when the impact energy is 20 J.

When the impact energy is 20J, the kinetic energy change history of the four models is shown in Figure 11. It can be seen from Figure 11 that when the punch contacts with the composite plate, the velocity gradually decreases to zero, and then it is ejected and detached from the composite plate. The kinetic energy when the punch separates from the plate is the residual energy of impact. According to the kinetic energy change curve in Figure 11 *.* The calculation results show that, as the fiber spiral angle decreases from 60° to 15°, the dissipative energy of sample 30 increases by 5.94% compared with that of sample 60, and the dissipative energy of sample 15 increases by 18.37% compared with that of sample 60. The sample 90 model consumes the most energy. According to the analysis results of the above four models, this is due to more matrix and fiber damage in sample 90, which dissipates more energy.

### Bionic specimens impact test

**FIGURE 12:**
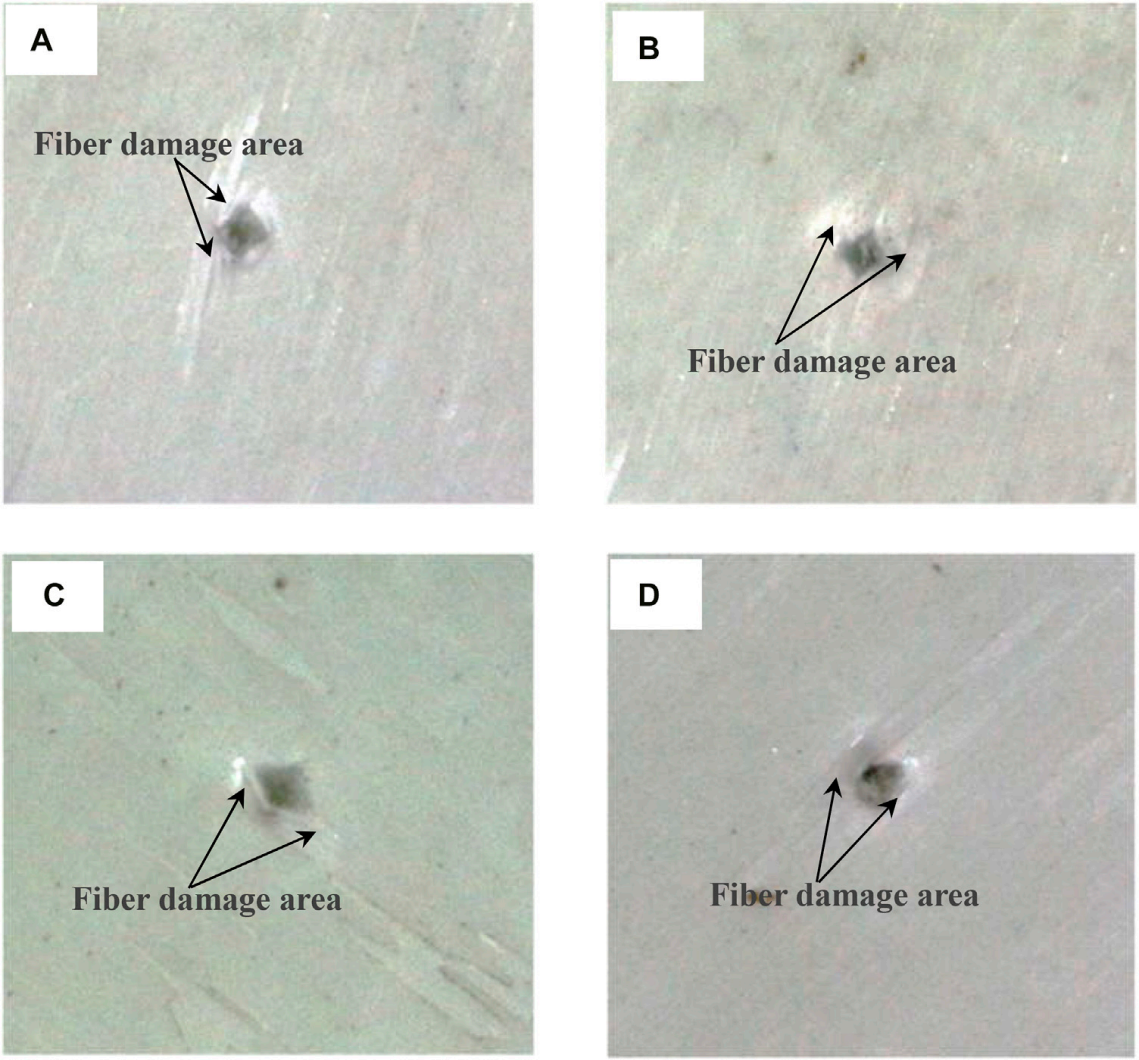
Impact damage of four biomimetic specimens. **(A)** Specimen15, **(B)** specimen30, **(C)** specimen60, **(D)** specimen90.

Figure 12 shows the failure mode of layered bionic specimens after impact. It is indicated that bionic composite laminates with different helix angles have varying degrees of damage under the same impact load. A total of 20 effective samples were tested in this impact test, with 5 samples of each type. According to the measurement results of punch impact depth of the four kind samples, the average impact depths of punch of Sample15, Sample30, Sample60 and Sample90 are 4.26mm, 4.11mm, 5.14mm and 6 mm respectively, Sample 90 was penetrated. In addition, based on the impact analysis results of four bionic composites under the same impact energy, namely, stress distribution (Figure 4), fiber compression failure (Figure 8), fiber tensile failure (Figure 10). It can be seen from the comprehensive analysis that the biomimetic composite model with helix angle of 30° has better comprehensive capacity of anti-impact damage. Thus, the impact damage area of the sample with 30° helix angle is smallest among the four types of bionic composites. It can be concluded that the bionic composite laminate with fiber helix angle of 30° has a better ability to resist impact damage, which is consistent with the results of finite element impact analysis, which also shows the correctness of the FE analysis.

The model for the FE simulation analysis is rectangular, and the sample for the drop-weight test is square, the geometric size of the sample will affect its mechanical behavior. Since the focus of this study is to compare and analyze the impact resistance of different fiber helix angles on the bionic composites, a unified geometric dimension is adopted in the FE analysis process, and the impact trend of this geometric dimension on the four bionic composites is similar. In the impact test, the test standard of ASTM-D-7136 was referred to. The geometric dimensions of the four test specimens are square, and the influence trend of the geometric dimensions on the mechanical behavior of the four impact specimens is similar. Therefore, we believe that geometric dimensions of the specimen in the FEM model and in the experiments are not the same, which will not significantly affect the research results of the mechanical behavior of the four bionic composites.

## Conclusion

In order to investigate the effect of fiber periodic helical structure on the impact characteristics of multilayer composites, four kinds of biomimetic composite models with different fiber helix angles were established based on the micro-nano structure of osteon, the impact characteristics and energy dissipation capacity of the four models were investigated. Then, the biomimetic structure with different helix angles were fabricated and tested. The conclusions as follows.

(1) The stress distribution and concentration of materials are affected by fiber helix angle. With the same material composition and material performance parameters, the stress concentration of composite materials under external impact can be effectively improved by adjusting the fiber arrangement method. The analysis results shown that the larger the fiber helix angle, the more serious the stress concentration phenomenon. And compared with the Sample30 model, the maximum stress of Sample60 and Sample90 models increases by 38.1% and 69.8%, respectively.
(2) The impact characteristics and energy dissipation capacity of multi-layer fiber reinforced composites are affected by the way of fiber laying. The fiber failure analysis results shown that among the four biomimetic composite models with fiber helix angles of 15°, 30°, 60° and 90°, the model with a fiber helix angle of 30° has the best resist impact damage. Moreover, in the case of without impact damage, the smaller the fiber helix angle, the more energy dissipated in impact process.
(3) The impact test results indicated that the impact damage area of the specimen with 30° helix angle is smallest among the four types of bionic specimens and has a better ability to resist impact damage, which is consistent with the results of FE impact analysis. Thus, the model with a fiber helix angle of 30° has the best comprehensive ability to resist impact damage.
(4) The helical structure of mineralized collagen fibers in osteon is the result of natural selection of biological evolution. This special structure can effectively improve the resist impact of cortical bone. The research results can provide useful guidance for the design and fabrication of high-performance biomimetic composites.

## Data Availability

The original contributions presented in the study are included in the article/Supplementary Material, further inquiries can be directed to the corresponding authors.

## References

[B1] Alizadeh S. R. Ebrahimzadeh M. A. (2022). O-substituted quercetin derivatives: Structural classification, drug design, development, and biological activities, a review. J. Mol. Struct. 1254, 132392. 10.1016/j.molstruc.2022.132392 34971873

[B2] Bhudolia S. K. Joshi S. C. (2018). Low-velocity impact response of carbon fibre composites with novel liquid Methyl methacrylate thermoplastic matrix. Compos Struct. 203, 696–708. 10.1016/j.compstruct.2018.07.066

[B3] Carnelli D. Vena P. Dao M. Ortiz C. Contro R. (2013). Orientation and size dependent mechanical modulation within individual secondary osteons in cortical bone tissue. J. R. Soc. Interface 10, 20120953. 10.1098/rsif.2012.0953 23389895 PMC3627101

[B4] Chang F. K. Lessard L. B. (1991). Damage tolerance of laminated composites containing an open hole and subjected to compressive loadings: Part I—analysis. J. Compos. Mater. 25 (1), 2–43. 10.1177/002199839102500101

[B5] Currey J. D. (2012). The structure and mechanics of bone. J. Mat. Sci. 47, 41–54. 10.1007/s10853-011-5914-9

[B6] Doddamani S. Kulkarni S. M. Joladarashi S. Kumar T S M. Gurjar A. K. (2023). Analysis of light weight natural fiber composites against ballistic impact: A review. Int. J. Lightweight Mater. Manuf, 1–41. 10.1016/j.ijlmm.2023.01.003

[B7] Dong Y. Wang F. Zhang Y. Shi X. Zhang A. Shuai Y. (2022). Experimental and numerical study on flow characteristic and thermal performance of macro-capsules phase change material with biomimetic oval structure. Energy 238, 121830. 10.1016/j.energy.2021.121830

[B8] Ekhtiyari A. Shokrieh M. M. (2022). A novel rate-dependent cohesive zone model for simulation of mode I dynamic delamination in laminated composites. Compos. Struct. 281, 114962. 10.1016/j.compstruct.2021.114962

[B9] Fratzl J. W. C. Fratzl P. (2010). Biological composites. Annu. Rev. Mater Res. 40, 1–24. 10.1146/annurev-matsci-070909-104421

[B10] Giner E. A. Arango C. Fuenmayor F. J. (2014). Influence of the mineral staggering on the elastic properties of the mineralized collagen fibril in lamellar bone. J. Mech. Behav. Biomed. Mat. 42, 243–256. 10.1016/j.jmbbm.2014.11.022 25498297

[B11] Giner E. Arango C. Vercher A. Javier-Fuenmayor F. (2014). Numerical modelling of the mechanical behaviour of an osteon with microcracks. J. Mech. Behav. Biomed. Mat. 37, 109–124. 10.1016/j.jmbbm.2014.05.006 24907671

[B12] Giraudguille M. M. (1988). Twisted plywood architecture of collagen fibrils in human compact bone osteons. Calcif. Tissue Int. 42 (3), 167–180. 10.1007/bf02556330 3130165

[B13] Gupta H. S. Stachewicz U. Wagermaier W. Roschger P. Wagner H. Fratzl P. (2006). Mechanical modulation at the lamellar level in osteonal bone. J. Mat. Res. 21 (8), 1913–1921. 10.1557/jmr.2006.0234

[B14] Hamed E. Lee Y. Jasiuk I. (2010). Multiscale modeling of elastic properties of cortical bone. Acta. Mech. 213, 131–154. 10.1007/s00707-010-0326-5

[B15] Hansen P. Martin R. D. C. B. (1999). 4ENF and MMB delamination characterization of S2/8552 and IM7/8552. London: European Research Office of the US Army.

[B16] Hashin Z. Rotem A. A. (1973). A fatigue failure criterion for fiber reinforced materials. J. Compos. Mater. 7 (4), 448–464. 10.1177/002199837300700404

[B17] Ingrole A. Aguirre T. G. Fuller L. Donahue S. W. (2021). Bioinspired energy absorbing material designs using additive manufacturing. J. Mech. Behav. Biomed. Mater. 119, 104518. 10.1016/j.jmbbm.2021.104518 33882409

[B18] Jansen M. A. Singh S. S. Chawla N. Franz N. M. (2016). A multilayer micromechanical model of the cuticle of *Curculio longinasus* Chittenden,1927 (Coleoptera: Curculionidae). J. Struct. Biol. 195, 139–158. 10.1016/j.jsb.2016.05.007 27189867

[B19] Jiang H. Ren Y. Liu Z. Zhang S. (2019). Microscale finite element analysis for predicting effects of air voids on mechanical properties of single fiber bundle in composites. J. Mater Sci. 54 (2), 1363–1381. 10.1007/s10853-018-2928-6

[B20] Karakuzu R. Erbil E. Aktas M. (2010). Impact characterization of glass/epoxy composite plates: An experimental and numerical study. Compos. Part B 41, 388–395. 10.1016/j.compositesb.2010.02.003

[B21] Liu D. Wagner H. D. Weiner S. (2000). Bending and fracture of compact circumferential and osteonal lamellar bone of the baboon tibia. J. Mater Sci. Mater Med. 11 (11), 49–60. 10.1023/a:1008989719560 15348099

[B22] Liu Y. Chen B. Yin D. (2017). Effects of direction and shape of osteocyte lacunae on resisting impact and micro-damage of osteon. J. Mater Sci. Mater Med. 28, 38. 10.1007/s10856-017-5850-6 28144850

[B23] Liu Y. Li A. Chen B. (2019). Effects of structure characteristics of osteocyte lacunae on squeeze damage resistance of osteons. Cells Tissues Organs 208, 142–147. 10.1159/000505135 32069449

[B24] Rahimizadeh A. Sarvestani H. Y. Li L. Robles J. B. Backman D. Lessard L. (2021). Engineering toughening mechanisms in architectured ceramic-based bioinspired materials. Mater. Des. 198, 109375. 10.1016/j.matdes.2020.109375

[B25] Reznikov N. Almany-Magal R. Shahar R. Weiner S. (2013). Three-dimensional imaging of collagen fibril organization in rat circumferential lamellar bone using a dual beam electron microscope reveals ordered and disordered sub-lamellar structures. Bone 52, 676–683. 10.1016/j.bone.2012.10.034 23153959

[B26] Reznikov N. Weiner S. (2014). Bone hierarchical structure in three dimensions. Acta Biomater. 10 (9), 3815–3826. 10.1016/j.actbio.2014.05.024 24914825

[B27] Rua J. Buchely M. F. Monteiro S. N. Echeverri G. I. Colorado H. A. (2021). Impact behavior of laminated composites built with fique fibers and epoxy resin: A mechanical analysis using impact and flexural behavior. J. Mater. Res. Technol. 21, 428–438. 10.1016/j.jmrt.2021.06.068

[B28] Sharma V. Borkute G. Gumfekar S. P. (2022). Biomimetic nanofiltration membranes: Critical review of materials, structures, and applications to water purification. Chem. Eng. J. 433 (3), 133823. 10.1016/j.cej.2021.133823

[B29] Varga P. Pacureanu A. Langer M. Suhonen H. Hesse B. Grimal Q. (2013). Investigation of the three-dimensional orientation of mineralized collagen fibrils in human lamellar bone using synchrotron X-ray phase nano-tomography. Acta Biomater. 9, 8118–8127. 10.1016/j.actbio.2013.05.015 23707503

[B30] Vercher A. Giner C. Arango C. Tarancon J. E. Fuenmayor F. J. (2014). Homogenized stiffness matrices for mineralized collagen fibrils and lamellar bone using unit cell finite element models. Biomech. Model Mechanobiol. 13 (2), 437–449. 10.1007/s10237-013-0507-y 23793930

[B31] Wang C. Su D. Xie Z. Wang H. Hazell P. J. Zhang Z. (2022). Dynamic behaviour of Bio-inspired heterocyclic aramid Fibre-reinforced laminates subjected to Low-velocity Drop-weight impact. Compos. Part A Appl. Sci. Manuf. 153, 106733. 10.1016/j.compositesa.2021.106733

[B32] Wang H. Wang C. Hazell P. J. Wright A. Zhang Z. Lan X. (2021). Insights into the high-velocity impact behaviour of bio-inspired composite laminates with helicoidal layups. Polym. Test. 103, 107348. 10.1016/j.polymertesting.2021.107348

[B33] Wang Y. Naleway S. E. Wang B. (2020). Biological and bioinspired materials: Structure leading to functional and mechanical performance. Bioact. Mater. 5 (4), 745–757. 10.1016/j.bioactmat.2020.06.003 32637739 PMC7317171

[B34] Weiner S. Arad T. Sabanay I. Traub W. (1997). Rotated plywood structure of primary lamellar bone in the rat: Orientations of the collagen fibril arrays. Bone 20 (6), 509–514. 10.1016/s8756-3282(97)00053-7 9177863

[B35] Xu J. Rho J. Y. Mishra S. R. Fan Z. (2003). Atomic force microscopy and nanoindentation characterization of human lamellar bone prepared by microtome sectioning and mechanical polishing technique. J. Biomed. Mater. Res. 67A (3), 719–726. 10.1002/jbm.a.10109 14613218

[B36] Yin D. Chen B. Lin S. (2021). Finite element analysis on multi-toughening mechanism of microstructure of osteon. J. Mech. Behav. Biomed. Mater. 117, 104408. 10.1016/j.jmbbm.2021.104408 33657473

[B37] Zhang Y. Chen P. (2021). An improved methodology of constructing inter-fiber failure criteria for unidirectional fiber-reinforced composites. Compos. Part A Appl. Sci. Manuf. 145, 106369. 10.1016/j.compositesa.2021.106369

